# Electroacupuncture at Acupoints Reverses Plasma Glutamate, Lipid, and LDL/VLDL in an Acute Migraine Rat Model: A **^1^**H NMR-Based Metabolomic Study

**DOI:** 10.1155/2014/659268

**Published:** 2014-01-28

**Authors:** Zishan Gao, Xuguang Liu, Shuguang Yu, Qi Zhang, Qin Chen, Qiaofeng Wu, Juan Liu, Bo Sun, Li Fang, Jia Lin, Bing-Mei Zhu, Xianzhong Yan, Fanrong Liang

**Affiliations:** ^1^Clinical Acupuncture and Moxibustion Department, Second School of Clinical Medicine, Nanjing University of Chinese Medicine, Nanjing, Jiangsu 210029, China; ^2^Acupuncture and Tuina College, Chengdu University of TCM, Chengdu, Sichuan 610075, China; ^3^National Center of Biomedical Analysis, Beijing 100850, China; ^4^The Third Affiliated Hospital of Zhejiang Chinese Medical University, Hangzhou, Zhejiang 310006, China; ^5^Department of Biochemistry and Molecular Biology, West China School of Preclinical and Forensic Medicine, Sichuan University, Chengdu, Sichuan 610041, China

## Abstract

*Background*. The objective of this study was to identify potential biomarkers of electroacupuncture (EA) on relieving acute migraine through metabolomic study. *Methods*. EA treatments were performed on both acupoints and nonacupoints on the nitroglycerin (NTG)-induced migraine rat model. NMR experiments and multivariate analysis were used for metabolomic analysis. *Results*. The number of head-scratching, the main ethology index of migraine rat model, was significantly increased (*P* < 0.01) after NTG injection. The plasma metabolic profile of model group was distinct from that of the control group. Glutamate was significantly increased (*P* < 0.01), whereas lipids were significantly decreased (*P* < 0.01) in model rats. After EA at acupoints, the metabolic profile of model rats was normalized, with decreased glutamate (*P* < 0.05) and increased lipids (*P* < 0.01). In contrast, EA at nonacupoints did not restore the metabolic profile, but with six metabolites significantly different from acupoints group. Interestingly, the number of head-scratching and glutamate level were significantly decreased (*P* < 0.05) after receiving EA at both acupoints and nonacupoints. *Conclusions*. EA at acupoints may relieve acute migraine by restoring the plasma metabolic profile and plasma glutamate, while EA at nonacupoints may modestly relieve acute migraine by decreasing plasma glutamate.

## 1. Introduction

Migraine is one of the most common neurological disorders characterized by recurrent unilateral, throbbing headaches and neurological dysfunction, with or without aura [1]. Approximately 16%–18% of women and 6%–8% of men in the USA suffer from migraine during the most productive years of their professional lives [2–4]. Although enhanced excitatory neurotransmitters such as glutamate (Glu), which facilitate spontaneous cortical spreading depression (CSD), are valued as central mechanism of triggering migraine, the pathophysiology of migraine is attributed to multiple factors, and many of these aspects are still not unraveled [5, 6]. Drug treatment with oral nonsteroidal anti-inflammatory drugs (NSAIDs) and triptans for relieving migraine usually have a modest effect and cause several side effects, such as gastrointestinal and cardiovascular disorders [7].

Acupuncture is a procedure whereby fine needles are inserted and manipulated into related acupoints of the individual, for the purpose of treating the disease. Although acupuncture has been widely used for migraine prophylaxis and treatment, little is understood about its biological mechanism. A 2009 Cochrane meta-analysis proclaimed acupuncture as safe and effective for migraine prophylaxis compared to prophylactic drug treatment [8]. The US Headache Consortium has also deemed acupuncture to be an important therapy for management of migraine [9]. Recently, an increasing number of studies have confirmed that mechanistically, acupuncture analgesia works through the release of opioid peptides in the central nervous system (CNS), which occurs in response to long-lasting activation of ascending sensory tracks stimulated by acupuncture manipulation [10–13]. However, the primary mechanism by which acupuncture relieves migraine has not been well established. Further, lately a series of high quality trials have fuelled suspicions that acupuncture at acupoints is no more effective than acupuncture at nonacupoints or sham acupuncture in reducing migraine headaches [14–17]. Hence, two urgent questions in the field of acupuncture for migraine have been raised. First, are there any neurological or metabolic biomarkers that provide supporting evidence for acupuncture to relieve migraine? Second, are there true neurological or metabolic differences between acupuncture at acupoints and acupuncture at nonacupoints?

To address these two questions, nitroglycerin-treated (NTG) rats are employed in this study as acute migraine model to detect metabolic changes of acute migraine treated by electroacupuncture. NTG rat model is regarded as a reliable animal model for acute migraine. It replicates neurogenic inflammation and hyperalgesia of human migraines, and it has been successfully established in different migraine studies [18–22].

Metabolomics has rapidly emerged as a powerful tool for identification of new biomarkers and for monitoring the dynamic pathophysiological metabolic changes of whole organisms in disease states [23]. Recent advances in nuclear magnetic resonance (NMR) spectroscopy, aided by data-reduction techniques, have facilitated the use of metabolomics as a direct functional readout of the pathological state from biofluids and tissues [24–26]. The power of metabolomics lies in monitoring systemic metabolite changes and characterizing complete metabolic profiling, which properly meet the essence of Traditional Chinese Medicine (TCM) and acupuncture for its sensitivity and complexity. Therefore, there are increasing studies that employ metabolomics to detect metabolic evidence for Traditional Chinese Medicine and acupuncture [27–30].

In the current study, we employed a developed ^1^H-NMR-based metabolomic technology to investigate metabolite changes in acute migraine evoked in NTG rat model and the effect of electroacupuncture on treating an acute migraine attack.

## 2. Methods

### 2.1. Ethics Statements

All experiments were conducted in strict accordance with the guidelines of International Association for the Study of Pain and Chinese national regulations for experimental animal use. The study was approved by the Sichuan Institutional Review Board for Animal Experiments (permit number: Sichuan experimental animal institute (97) 6). *All surgeries were performed under sodium pentobarbital anesthesia, and all efforts were made to minimize suffering.*

### 2.2. Acute Migraine Model

**Figure 1:**
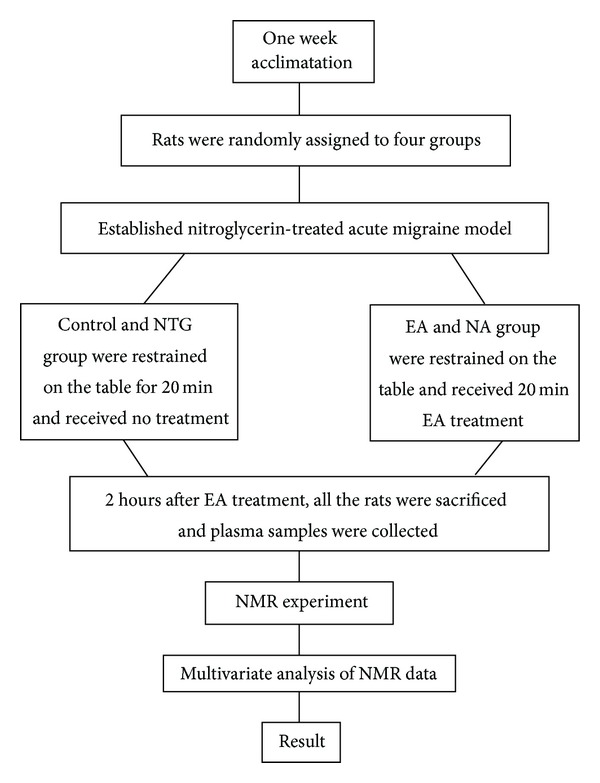
Trial fluidogram.

The overall experimental approach is shown in Figure 1. Fifty female *Sprague Dawley* rats, 3 months old and weighing 160 ± 20 g, were purchased from the Experimental Animal Center of Chengdu University of Traditional Chinese Medicine. Rats were housed in a well-ventilated colony room at a temperature of 15–22°C and a relative humidity of 50–70% on a 12 h light/12 h dark cycle. Food and tap water were provided *ad libitum*. Rats were allowed to acclimatize for one week before experiments began and then were randomly divided into 4 groups using an SPSS randomization process: control (untreated, without any treatment), NTG (nitroglycerin-treated model), EA (electroacupuncture at acupoints following NTG-treatment), and NA (electroacupuncture at nonacupoints following NTG-treatment). Each group has 10 rats and was fed separately in two metabolic cages.

All rats were restrained on an operating table for further treatment. The rats in control group received no injection, while other three groups were injected intraperitoneally with NTG (10 mg/kg of 2 mg/mL solution [18]) according to previous study [22]. Thirty minutes after injection, three groups of NTG rats displayed red ear, frequent head-scratching, and climbing cage, which represent the anxiety and discomfort, indicating the successful establishment of migraine model [18]. These typical symptoms lasted for 4 hours, which is consistent with previous reports. In contrast, the control rats did not show these symptoms.

Following NTG injection, the behavior of the rats was observed and documented. The total time of ethology observation lasted for 4 h, and each 30 min of observation was considered a time interval. The number of head-scratching was quantitatively measured as the main ethology index [18–22].

### 2.3. Electroacupuncture Treatment

One hour after injection, animals were restrained on an operating table for electroacupuncture treatment. The EA group received filiform needles that were inserted perpendicularly and bilaterally to a depth of 2-3 mm at the points of SJ5 (Waiguan, located on lateral side of forefoot, 3 mm away from wrist joint and in the middle of ulnar bone and radial bone) and GB34 (Yanglingquan, located on the postlateral side of knee joint, below capitulum fibulae) along the Shao Yang meridian of Hand and Foot (see Supplementary Figure 1 available online at http://dx.doi.org/10.1155/2014/659268). These acupoints are most frequently used to relieve migraine attacks [31]. Nonacupoints were located 5 mm above from each acupoint according to previous studies (Supplementary Figure 1) [14].

Transcutaneous electroacupuncture (EA) stimulation was then conducted at each acupoint for 20 min [32] using a Han's acupoint nerve stimulator (HNAS-200, Nanjing, China). Stimulation frequency was set at 14 Hz, and intensity was increased from 0.1 mA to a maximum of 1.0 mA until the rats' feet began to tremble slightly [32]. For the NA group, an identical electroacupuncture procedure was conducted, except that needles were placed 5 mm above from each acupoint [31]. Animals in the control and NTG groups were also fixed on the table for 20 min, but no electroacupuncture was performed. All rats were conscious during treatment.

### 2.4. Sample Collection and Pretreatment

To avoid potential acute effects of EA, rats were anesthetized with 3% sodium pentobarbital (40 mg/kg., i.p.) at 2 hours after electroacupuncture treatment [32] and then sacrificed at 4 hour after nitroglycerin injection by exsanguination from the femoral artery. Blood (~3 mL) from the femoral artery was collected into a heparin sodium tube, placed on ice for 30 minutes, and then centrifuged (3000 ×g, 4°C, 15 min). Plasma was collected and stored at −70°C.

Before NMR analysis, plasma samples were thawed, centrifuged (13000 ×g, 4°C, 10 min), and the supernatant extracted. In 5 mm NMR tubes, 300 *μ*L of each supernatant sample was mixed with 250 *μ*L of D_2_O to lock the field frequency and 50 *μ*L of 3-trimethylsilyl-2H_4_-propionic acid sodium salt (TSP) in D_2_O (1 mg/mL) as a chemical shift reference. All samples contained a final volume of 600 *μ*L and were vibrated repeatedly [33, 34].

### 2.5. NMR Experiments

^1^H NMR spectra of plasma were acquired with a Varian INOVA 600 MHz NMR spectrometer at 27°C using a Carr-Purcell-Meiboom-Gill (CPMG) spin-echo pulse sequence with a total spin-spin relaxation delay (2 n*τ*) of 320 ms. Water suppression was achieved with an irradiation of the water peak during the recycle delay (2 s) and mixing time (*t*_*m*_) of 150 ms. Free induction decays (FIDs) were collected into 32,000 data points with a spectral width of 8,000 Hz over 64 scans. Presaturate frequency and central frequency were equally on the water peak. The FIDs were zero failed by a factor of two and multiplied by an exponential line-broadening factor of 0.5 Hz prior to Fourier transformation. In addition, diffusion-edited experiments were also performed with bipolar pulse pair-longitudinal eddy current delay (BPP-LED) pulse sequence. The gradient amplitude was set at 35.0 G/cm with a diffusion delay of 100 ms. A total of 128 transients and 16,000 data points were collected with a spectral width of 8,000 Hz. A line-broadening factor of 1 Hz was applied to FIDs before Fourier transformation.

^1^H NMR spectra of plasma were manually phased and baseline-corrected using VNMR 6.1C software (Varian Inc.). For CPMG spectra, each spectrum over the range of *δ* 0.4–4.4 was data-reduced into integrated regions of equal width (0.01 ppm). For BPP-LED data, each spectrum over the range of *δ* 0.1–6.0 was segmented into regions of equal width (0.01 ppm). The regions containing the resonance from residual water (*δ* 4.6–5.1) were excluded. The integral values of each spectrum were normalized to a constant sum of all integrals in a spectrum to reduce any concentration plot variations between samples. Identification of metabolites in the spectra was accomplished based on information in the literature and the Chenomx NMR Suite 4.5 (Chenomx, Calgary, Canada).

**Figure 2:**
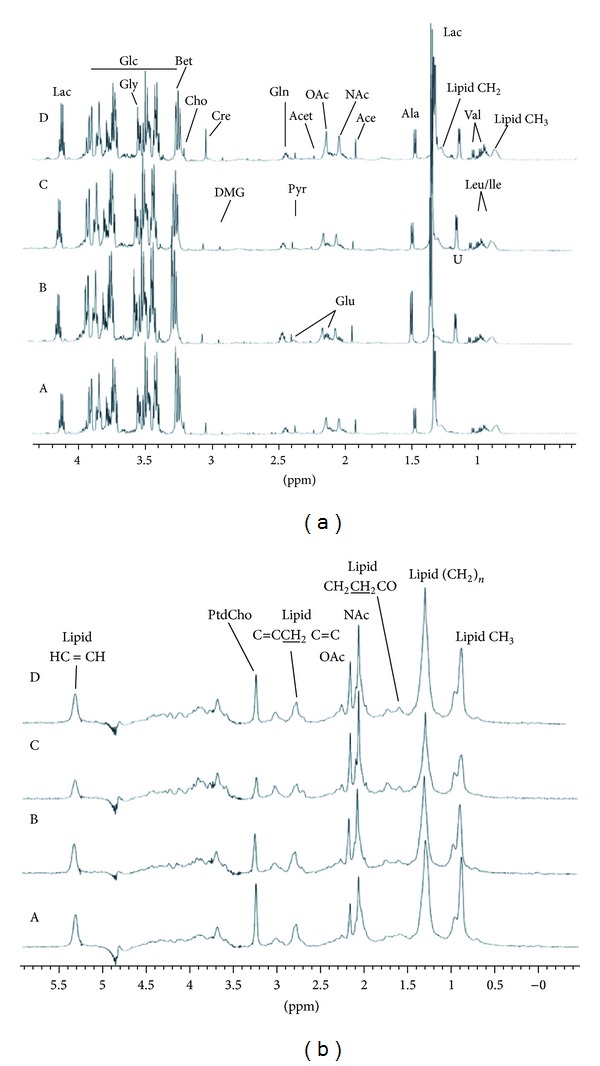
^1^H NMR spectra of rat plasma samples. (a) CPMG spectra; (b) LED spectra; A, control; B, NTG group; C, NA group; D, EA group (*n* = 10).

Based on previous studies, the HMDB website (http://www.hmdb.ca/) and the ChenomxNMR Suite software, major metabolites in plasma were identified and shown in Figure 2. CPMG pulse sequence was used to emphasize the resonances of small metabolites in plasma, while resonances from macromolecules were attenuated (Figure 2(a)).  Figure 2(b) shows diffusion-edited NMR spectra of plasma from each group, displaying the signals of lipids, N-acetyl glycoproteins (NAc), and O-acetyl glycoproteins (OAc) groups of glycoproteins. Subtle differences of these spectra were observed by visual inspection among the four groups. Further analysis was performed using multivariate statistical analysis to determine the metabolic changes among the four groups.

### 2.6. Pattern Recognition and Statistics

The resulting integral data were imported into SIMCA-P (version 10.04; Umetrics, Umeå, Sweden) for multivariate analysis. Prior to analysis, the CPMG data were mean-centered and Pareto-scaled, and the BPP-LED data were mean-centered. CPMG data and LED data were both subjected to principal component analysis (PCA) and partial least square discriminate analysis (PLS-DA) to discriminate differentiation in metabolites among the groups. PCA was firstly performed on the normalized ^1^H NMR dataset after Pareto scaling in this study. Data were visualized by using the principal component (PC) score and loading plots. Each point on the scores plot represents an individual sample, and each point on the loadings plot represents a single NMR spectral region. Orthogonal signal correction (OSC) was further used to remove the variations not correlated to group membership and to maximize the separation, followed by PLS analysis. The final lists of metabolites were chosen on the basis of the variables of importance parameter (VIP), which is a measure of each variable's relative influence on the model [35]. Because in this study we found series of metabolite changes and they were correlated with each other among metabolic systems, therefore multivariate analysis of variance was performed to discriminate significant changes of metabolites identified by SIMCA-P. Ethology data were calculated by repeated measures analysis of variance and multivariate analysis of variance.

## 3. Results

### 3.1. Electroacupuncture at Both Acupoints and Nonacupoints Reduce Head-Scratching Number in NTG Rats

Initially, the quantitative ethology observations, for the purpose of detecting NTG-induced acute migraine rat model and the effect of EA, were recorded and calculated. Following NTG injection, the number of head-scratching in the NTG group, EA group, and NA group increased significantly from NTG injection to 60 min compared to the control group (*P* < 0.01) (Figure 6(a)). Specially, the number of head-scratching in the NTG group significantly increased from NTG injection to 60 min and from 120 min to 240 min, indicating that a successful rat model of acute migraine had been established [21]. Secondly, 20 min of EA at acupoints and EA at nonacupoints treatments were performed accordingly on EA and NA groups from 60 min to 120 min after NTG injection. Subsequently, the number of head-scratching was obviously decreased (*P* < 0.05) in both EA and NA group from 120 min to 240 min (Figure 6(a)) compared to NTG group, suggesting that a typical symptom of acute migraine was relieved by performing EA at both acupoints and nonacupoints. In addition, the number of head-scratching was significantly decreased (*P* < 0.05) in the NA group from 210 min to 240 min following NTG administration (Figure 6(a)) compared to EA group. In conclusion, these data showed that the number of head-scratching in NTG group was significantly increased (*P* < 0.01) compared to the control group, while the number of head-scratching in EA and NA group was significantly decreased (*P* < 0.05) relative to NTG group after receiving EA at both acupoints and nonacupoints.

### 3.2. NMR Metabolic Profiling Can Differentiate Acute Migraine Rat Model

**Figure 3:**
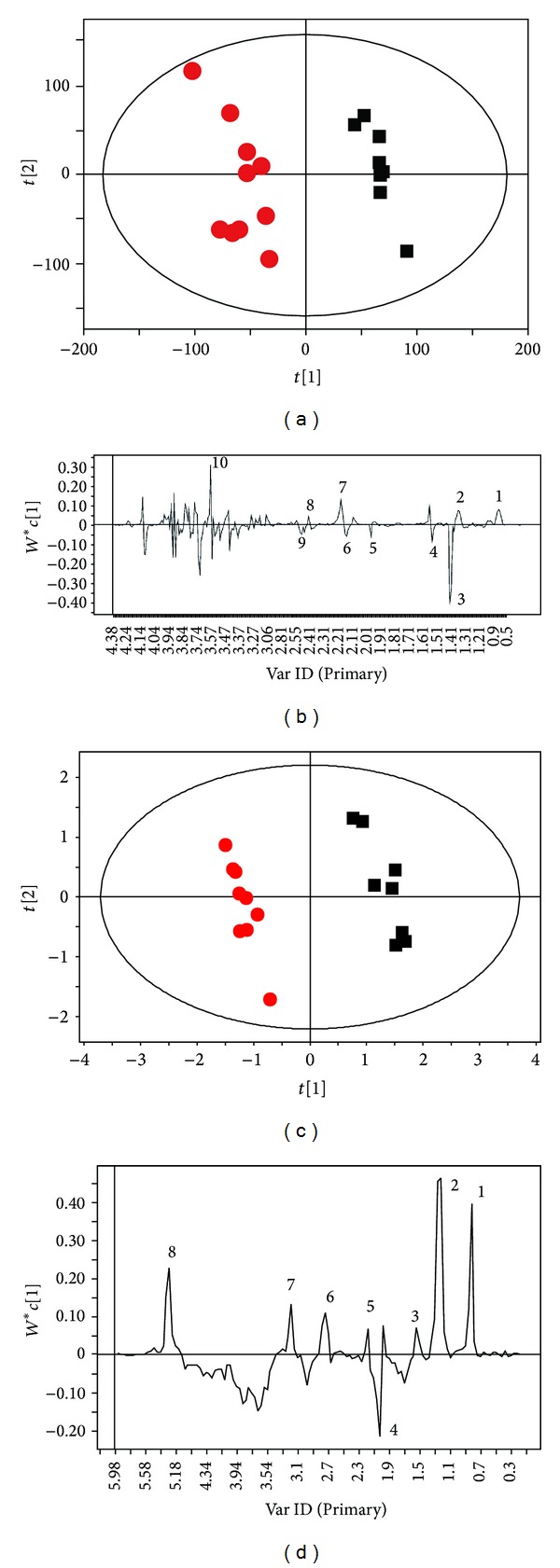
Clear separation of CPMG and LED spectra between NTG (red dots) and control (black boxes) groups. (a) Scores plot of OSC-PLS analysis of CPMG data; (b) corresponding loadings line plot. (c) Scores plot of OSC-PLS analysis of LED data; (d) corresponding loadings line plot. Keys to B: (1) lipid CH_3_; (2) lipid CH_2_; (3) lactate; (4) alanine; (5) acetate; (6) glutamate; (7) OAc; (8) pyruvate; (9) glutamine; (10), glycine. Keys to D: (1) lipid CH_3_; (2) lipid CH_2_; (3) lipid CH_2_CH_2_CO; (4) NAc; (5) lipid CH_2_CO; (6) PUFA; (7) PtdCho; (8) lipid CH=CH (*n* = 10).

After NMR experiment, 22 metabolites in plasma of animal groups were identified by using Chenomx NMR Suite software and shown in Figure 2. To detect the role of electroacupuncture on metabolite changes in acute migraine rats, we first asked whether NMR metabolic profiling can differentiate between acute migraine rat model and untreated control rats. Thus, we performed PCA and PLS-DA with OSC pretreatment on the normalized ^1^H NMR CPMG dataset. A clear separation was achieved predominately along PC1 (Figure 3(a); *R*2*X* = 62.7%, *R*2*Y* = 99.1% and *Q*2 = 97.5%) between the control group and the NTG group. Based on the results of OSC-PLS-DA and multivariate analysis of variance, the dominant metabolites that influenced the differentiation between the control and NTG groups are displayed in the corresponding loadings plots (Figure 3(b)), the concentration plot (Figure 6(b)), and Table 1. Notably, NTG group demonstrated a significantly higher level of glutamate (*P* = 0.009), which is an important excitatory neurotransmitter for triggering migraine [36], and significantly lower levels of LDL/VDL (*P* = 0.001) and lipid (*P* = 0.008) compared with the controls. Moreover, PCA, PLS analysis with OSC pretreatment were also performed on the ^1^H NMR LED dataset, which is similar to the CPMG procedure. The results illustrated an obvious separation between the controls and the NTG group along PC1 (Figure 3(c); *R*2*X* = 53.1%, *R*2*Y* = 99%, *Q*2 = 96.3%), as shown in Table 2 and Figure 6(b). Together, we extracted an enhanced anaerobic glycolysis and reduced gluconeogenesis from the metabolic profiling of nitroglycerin-treated acute migraine rat model, as displayed by decreased glucose, pyruvic acid and increased lactic acid, alanine, and 3-hydroxybutyric acid (3-HB) (Figure 6(b)). Meanwhile, the enhancement of lipid metabolism was also demonstrated in the NTG acute migraine rat model, as indicated by decreased levels of lipid, unsaturated lipid (UFA), choline, and phosphatidylcholine (Ptdcho) (Figure 6(b)). The metabolite change at 1.14 ppm remains unidentified, but it seems unlikely that this metabolite could be a component of the NTG solution that would have influenced any group differences.

### 3.3. Electroacupuncture at Acupoints Reverses Plasma Metabolite Changes in the Acute Migraine Rat Model

**Figure 4:**
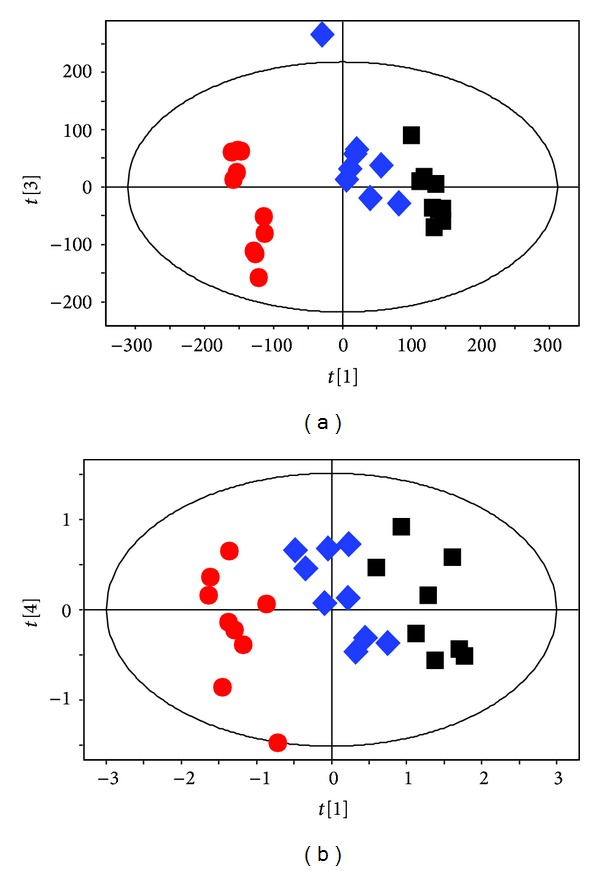
OSC-PLS analysis manifest EA at acupoints reverses plasma metabolite changes in the acute migraine rat model. NTG group (red dots), EA group (blue diamonds), and control group (black boxes). CPMG analyses were conducted (a) among three groups. LED analyses were conducted (b) among three groups (*n* = 10).

After establishing the metabolic profiling of NTG acute migraine rat model, we next determined whether EA reverses metabolite changes in the acute migraine rat model. We performed PLS analyses with OSC pretreatment on data from three groups: control, NTG, and rats who received EA at acupoints. After 20 min of EA at acupoints, we observed that the metabolic profiling of the EA group was similar to that of the control group, and both these groups were clearly separated from the NTG group along PC1 (Figure 4(a); *R*2*X* = 77.4%, *R*2*Y* = 98.3%, and *Q*2 = 92.6%). Interestingly, the EA group illustrated a significant lower level of glutamate (*P* = 0.048) and significant higher levels of LDL/VDL (*P* = 0.002) and lipid (*P* = 0.002) compared with the NTG group, which is the opposite of the metabolite changes in NTG group relative to controls (Figures 6(d) and 6(e)). Meanwhile, the concentration plot showed that the levels of lactic acid, OAc, pyruvic acid, alanine, LDL/VLDL, glutamate, glutamine, creatine, and lipid in EA group were restored to levels similar to that of the control group (Figures 6(c)–6(e)). Furthermore, the diffusion-edited NMR spectra was performed, which is similar to the CPMG procedure. The result demonstrated a clear differentiation among the control, NTG, and EA groups along PC1 (Figure 4(b); *R*2*X* = 80.9%, *R*2*Y* = 99.3%, *Q*2 = 88.7%), as shown in Figure 4(b). In the EA group, we found significantly higher levels of lipid (CH_2_), NAc and lower levels of choline (*P* < 0.05) compared with the NTG group (Figures 6(c) and 6(d)). Taken together, these data suggest that EA reverses NTG induced changes by restoring the metabolic profiling and reversing the levels of plasma metabolites, such as lactic acid, OAc, NAc, pyruvic acid, alanine, LDL/VLDL, glutamate, isoleucine, creatine, lipid (CH_2_), and lipid (CH_3_) in the EA groups relative to the NTG group.

### 3.4. Clear Separation of Metabolic Profiling between EA at Acupoints and EA at Nonacupoints

**Figure 5:**
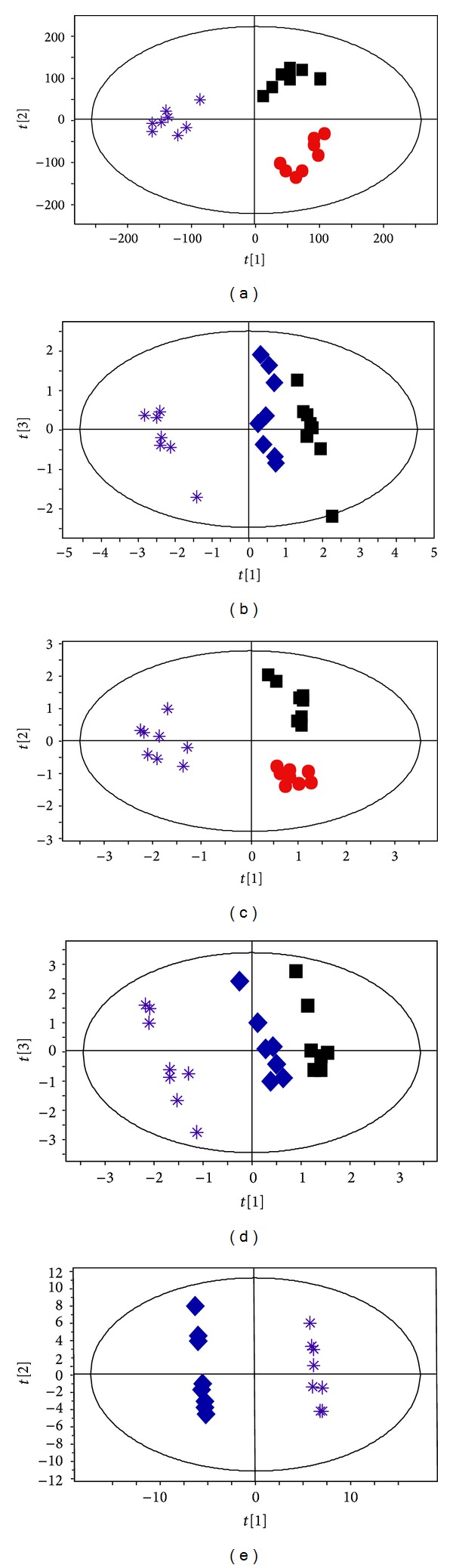
OSC-PLS analysis discriminates clear separation between EA at acupoints and EA at nonacupoints in the acute migraine rat model. NTG group (red dots), EA group (blue diamonds), NA group (purple stars), and control (black boxes) group. CPMG analyses were conducted (a) among three groups and (b) among EA, NA, and control group. LED analyses were conducted (c) among three groups and (d) among EA, NA, and control group and (e) between EA and NA group (*n* = 10).

**Figure 6:**
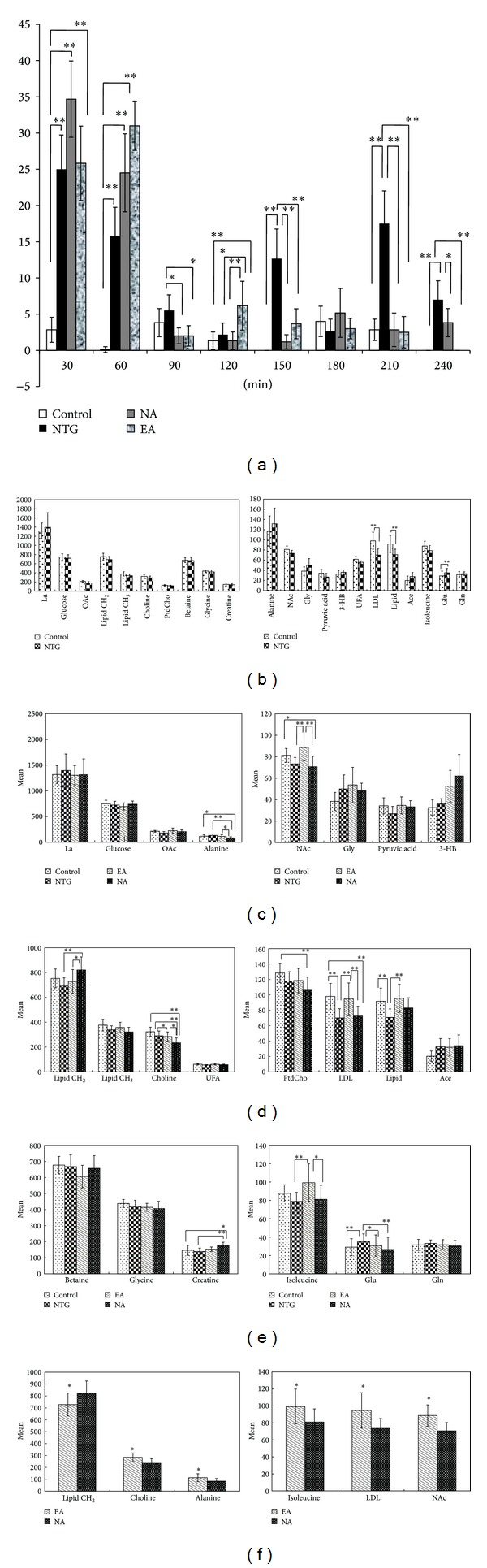
The number of head-scratching and the concentration of metabolites from control, NTG, EA, and NA groups. Repeated measures analysis of variance and multivariate analysis of variance were accordingly conducted among four groups. Each column represents the mean, with the SEM shown by error bars. (a) The number of head-scratching from control, NTG, EA, and NA groups after NTG injection (*n* = 6). *P < 0.05, **P < 0.01. (b) Differences in plasma levels of 22 metabolites were detected in nitroglycerin-induced rats compared with controls. LDL/VLDL, lipid were drastically decreased and glutamate was significantly increased in NTG group relative to control group (*n* = 10). **P < 0.01. (c) The metabolites changes of glycometabolism among control, NTG, EA, and NA group. EA group reversed plasma metabolite changes in NTG group (*n* = 10). *P < 0.05, **P < 0.01. (d) The metabolite changes of lipid metabolism among four groups (*n* = 10). *P < 0.05, **P < 0.01. (e) The metabolite changes of amino acids metabolism among four groups (*n* = 10). *P < 0.05, **P < 0.01. (f) Significant differences of metabolite changes between EA and NA group (*n* = 10). *P < 0.05.

A remaining question is whether there are any metabolomic differences between EA at acupoints and EA at nonacupoints. To address this question, we next executed PLS analysis with OSC pretreatment on both CPMG and LED data among the control, NTG, and the NA groups. After 20 min of EA at nonacupoints, we observed that the metabolic profiling of the NA group was clearly separated from that of the control and the NTG group (Figure 5(a), *R*2*X* = 74.2%, *R*2*Y* = 98.9%, and *Q*2 = 97.6%). Meanwhile, of particular interest are data shown in Figure 5(b) (*R*2*X* = 65.1%, *R*2*Y* = 99.1%, *Q*2 = 96.4%) and Figure 5(d) (*R*2*X* = 72.7%, *R*2*Y* = 98.8%, *Q*2 = 88.7%), illustrating that the metabolic profile of the NA group was clearly distinct from controls, unlike EA group, which was very similar to controls. Furthermore, a clear separation was observed between the EA group and the NA group along PC1 (Figure 5(e), *R*2*X* = 63.6%, *R*2*Y* = 99.9%, *Q*2 = 99.3%). The levels of six metabolites including lipid (CH_2_), choline, alanine, isoleucine, LDL/VLDL, and NAc were significantly different in the EA group compared to the NA group (Figure 6(f); *P* < 0.05). Combined, these data suggest that EA at nonacupoints cannot reverse NTG-induced changes, but there are distinct differences compared to EA at acupoints, and this was characterized by different metabolic profiling and a series of significant changes of plasma metabolites in the NA groups relative to EA group. In contrast, we found that the level of glutamate, an important excitatory neurotransmitter that triggers migraine, was significantly decreased in both EA and NA groups compared with the NTG group. This observation indicates that glutamate may be crucial for electroacupuncture to relieve acute migraine, but it is not acupoint-dependent.

## 4. Discussion

Our study found that EA at acupoints not only restores the metabolic profiling but also reverses a series of significant metabolite changes such as glutamate, LDL/VLDL, and lipid in the NTG-treated acute migraine model. In contrast, we found that EA at nonacupoints did not restore the metabolic profiling of NTG acute migraine rat. Six metabolites that might contribute to the specific effect of acupoints were identified by significant difference between EA at acupoints and EA at nonacupoints. Specially, we found that EA at both acupoints and nonacupoints may relieve acute migraine by decreasing the plasma intensity of glutamate. These data, to our knowledge, for the first time provide potential metabolomic evidence for the effect of EA on relieving acute migraine.

The NTG rat is a reliable animal model of acute migraine and was therefore employed in this study to determine the metabolic profile of acute migraine and the effect of electroacupuncture [18–22]. In the series of discomfort symptoms exhibited by NTG rats after being administered with nitroglycerin, “head-scratching,” which resembles the attack of human migraine, was quantitatively measured as an important observation index in acute migraine rat model [21]. The number of head-scratching obviously increased (*P* < 0.01) after NTG injection and lasted for 4 h, manifesting the key signal of success of acute migraine model.

The advantages of this study include the developed metabolomic strategy and advanced multivariate statistical analysis such as OSC-PLS-DA and MNOVA. Using a combination of these techniques, not only the whole plasma metabolic profiling was characterized, but also significant plasma metabolite changes were confirmed from all of small plasma metabolites changes of acute migraine rats. To investigate metabolic evidence of electroacupuncture relieving migraine, the metabolic profiling and the metabolic disturbances in NTG acute migraine rats were firstly presented in this study (Supplementary Figure 2). Glutamate, LDL/VLDL, and lipid were drawn out from all the metabolites changes and confirmed to be significantly reversed in the NTG-induced acute migraine animals.

As studies to date, glutamate plays a key role in the pathophysiology of migraine in both animal and human researches [36]. Ramadan found that glutamate facilitates cortical spreading depression (CSD), central sensitization, which are the central mechanisms for triggering migraine [37]. Peres et al. have shown that plasma glutamate levels are positively correlated with headache intensity in chronic migraine patients [38, 39]. Accordingly, our study further demonstrates that the elevation of glutamate in the plasma is a crucial contributory factor in acute migraine attack. On the other hand, recent migraine studies have revealed that normal weight migraine patients whose blood was collected on the eighth day after their last migraine attack presented an atherogenic lipid profile, including high density of LDL/VLDL [40]. In contrast, we found that LDL/VLDL and lipid were significantly lower in the acute migraine rat model. We hypothesize that the conflict between our results and those of the clinical study can be attributed to the use of nitroglycerin in the rat model. Nitroglycerin liberates nitric oxide (NO), which is known as endogenous vasodilator, thereby causing dilation of meningeal blood vessels [41–43]. There are increasing evidences to support that migraine attack is associated with dilatation of both extra- and intracranial vessels. Accordingly, one of the advantages of NTG-induced acute migraine model just lies in this dilation of meningeal blood vessels which identically occurred during migraine attack [44–47]. These dilated vessels induced by NTG may quickly increase the blood volume and therefore dilute the level of lipid and LDL/VLDL. Thus, the significantly lower LDL/VLDL and lipid levels might be induced by nitroglycerin injection. Although the significant changes in the lipid and LDL/VLDL may not be a possible biomarker of migraine owing to the inconsistence between NTG-induced rat model and clinical migraine patients, little is known about the change in lipid metabolism during acute migraine attack. Our results may provide a useful clue for clinical studies of acute migraine.

While acupuncture has been proved to be safe and effective in various high quality trials, the analgesic effect of acupuncture in a clinical setting for migraine is still a hotly debated issue. Based on a series of clinic studies that showed that acupuncture at acupoints was no more effective than acupuncture at nonacupoints, some authors wondered if the effect of acupuncture in relieving migraine is only due to the placebo effect and bias [48, 49]. Recently, a meta-analysis including 18,000 randomized patients in high-quality randomized control trails (RCTs) manifested novel evidence that acupuncture is superior to sham acupuncture and placebo for managing chronic pain, including migraine [49]. Accordingly, the increased number of head-scratching in NTG-injected rats was significantly reduced after receiving EA treatment in our study, suggesting that EA relieved acute migraine. However, the scientific mechanism addressing the analgesic effect of acupuncture for migraine is still not well established. Our study is at the first to confirm the potential metabolic evidence of EA relieving migraine for the following two metabolic mechanisms: (i) EA at acupoints can reverse the metabolic profiling of acute migraine rats; (ii) we found that these metabolites change significantly in the plasma of acute migraine rat model, including glutamate, LDL/VLDL, and lipid, which were significantly reversed and restored in acute migraine rats after receiving EA at acupoints (Figures 6(d) and 6(e)). Noteworthy, glutamate, which is crucial in triggering migraine, was decreased after EA treatment, providing direct metabolic evidence that EA relieves acute migraine. To date, increasing numbers of neurological studies have noted that elevated plasma glutamate levels are positively correlated with cortical neuronal hyperexcitability and increased levels of glutamate in the central nervous system (CNS) [39]. Enhanced glutamate released in brain tissue, induced by brief pluses of high K^+^, was found to causatively facilitate CSD, thereby triggering migraine [37]. To conclude, the increased plasma level of glutamate has been proved to be crucial for triggering acute migraine attacks through stimulating cortical neuronal hyperexcitability, increasing glutamate levels in the CNS, and facilitating CSD. Thus, the depletion of glutamate stimulated by EA could inhibit cortical neuronal hyperexcitability, lower glutamate levels in the CNS, depress CSD, and subsequently decrease acute migraine attack. In addition, acupuncture was illustrated to improve the blood flow and change blood perfusion in the previous studies [50, 51]. Hence, the significantly reversed LDL/VLDL and lipid after EA at acupoints in NTG rats may be attributed to the adjustment of blood flow and blood perfusion stimulated by EA at acupoints. In conclusion, the analgesic effect of EA for acute migraine may rely on substance metabolic basis rather than bias in this study. Therefore, the advantage underlying our results highlights the potential value of metabolomics for becoming surrogate outcome measure of further clinical acupuncture trials.

A series of high quality trials have found that acupuncture on acupoints was not superior to acupuncture on nonacupoints in reducing migraine headaches [14–17, 49, 52]. Consistent with these results, our study found that the number of head-scratching was significantly decreased after both EA at acupoints and EA at nonacupoints treatments. In particular, we found that plasma glutamate levels significantly decreased after performing EA at both acupoints and nonacupoints. It is important to note that this adjustment provides potential evidence that both EA at acupoints and EA at nonacupoints treatments may relieve acute migraine by decreasing the intensity of plasma glutamate. In contrast, Yang et al. reported that the different brain regions related to pain were evoked by performing EA at different acupoints through advanced PET-CT technology [53]. In comparison with previous studies, our findings firstly discriminate significant differences that may contribute to the specific effect of acupoints relative to nonacupoints by two mechanisms: (i) a clear difference between the metabolic profiling of EA and NA groups was successfully demonstrated (Figure 5(e)). (ii) Levels of six metabolites were found to be significantly different between EA and NA groups (Figure 6(f), *P* < 0.05). Furthermore, we found that the metabolic profile induced by EA at nonacupoints is significantly different from that induced in controls and NTG-induced rats. The significant change in lipid CH_2_ and PtdCho compared to the control and NTG-induced rats may be an important contributor for this distinct metabolite profile. Whereas, the concentration plot and metabolic profile showed that EA at acupoints can reverse metabolites which were significantly changed in acute migraine model, but EA at nonacupoints did not show such an effect. In light of these facts, the effect of EA at acupoints, but not EA at nonacupoints, has been suggested to be more feasible for adjusting the metabolic disturbance in the plasma of acute migraine.

### 4.1. Limitations and Directions for Future Studies

The time-related metabolomic changes of electroacupuncture were not detected in this study. Acupuncture is usually performed in one session during the clinical treatment of acute migraine, and our study is consistent with such session. As numerous studies have granted the sensitivity and accuracy of metabolomics for assessing the pathological states of diseases, our study may provide direct and accurate readout of the metabolic changes for electroacupuncture relieving acute migraine. We have already conducted a dynamic randomized control trial to investigate the effects of electroacupuncture on migraine induced metabolic changes in migraine patient (unpublished). Future studies will extend this work using a larger group of clinical migraine patients.

## 5. Conclusion

In summary, EA at acupoints may relieve acute migraine through restoring metabolic profile and reversing significant metabolite changes such as glutamate in acute migraine rats, while EA at nonacupoints might modestly relieve acute migraine by decreasing the intensity of plasma glutamate.

## Supplementary Material

Supplementary Figure 1: Anatomy of Rat Acupoints. Acupuncture treatment on NTG rats were conducted according to the picture of anatomy of rat acupoints. 15, Yanglingquan (GB34); 30, Waiguan (SJ5); 43-44, non-acupoints.
Supplementary Figure 2: Imbalanced metabolism systems in NTG group. Imbalanced glycometabolism, lipid metabolism and amino acids metabolism were illustrated between NTG and control group.

## Figures and Tables

**Table 1:** Changes of plasma metabolites in CPMG NMR spectra induced by NTG and the effects of EA.

Metabolites	Peak regions (*δ*)	NTG versus untreated control	NTG versus EA	NTG versus NA
Unknown	1.14, 1.18	↑	↓	—
Lactic acid	1.34, 4.1	↑	↓	↓
Glutamate	2.11, 2.35	↑	↓	↓
Glutamine	2.42	↑	↓	↓
Glycerine	3.62, 3.66	↑	↓	↑
OAc	2.14, 2.18	↓	↑	↑
LDL/VLDL	0.86	↓	↑	—
Glycine	3.54	↓	↑	—
NAc	2.02, 2.06	↓	↑	—
Betaine	3.26	↓	↑	—
Lipid	1.26, 1.3	↓	↑	↑
D-Glucose	3.42, 3.38, 3.46, 3.5, 3.74, 3.82, 3.86,	↓	↑	↓
Alanine	1.46	↑	↑	↓
L-Isoleucine	0.94	↓	↑	—
Choline	3.22	↓	↑	↓
Pyruvic acid	2.38	↓	↑	—
Acetoacetic acid	1.9, 1.94	↑	↓	—
3-HB	1.22	↑	↓	↑
Creatine	3.94	↓	↑	↑

NAc: N-acetyl glycoproteins; OAc: O-acetyl glycoproteins; 3-HB: 3-hydroxybutyric acid. The arrows indicate the direction of the change (↑: increase;

↓: decrease) in the concentration among untreated control group of animals, EA group of animals, and NA group of animals relative to NTG group animals (*n* = 10).

**Table 2:** Changes of plasma metabolites in LED NMR spectra induced by NTG and the effects of EA.

Metabolites	Peak regions (*δ*)	NTG versus untreated control	NTG versus EA	NTG versus NA
NAc	2.02, 2.06	↑	↓	↑
OAc	2.14, 2.18	↑	↓	↑
Lipoid	1.3, 1.34, 1.26, 2.22, 2.26	↓	↑	↓
Unsaturated lipid	5.26, 5.34	↓	↑	↓
LDL/VDL	0.86, 0.9	↓	↑	↑
PUFA	2.74, 2.78, 2.82	↓	↑	—
PtdCho	3.22	↓	↑	↓
FA	1.58, 1.74, 1.78, 1.7, 1.82, 2.26	↓	↑	↑

NAc: N-acetyl glycoproteins; OAc: O-acetyl glycoproteins; PtdCho: phosphatidyl choline; UFA: unsaturated lipid; FA: fatty acid; PUFA: polyunsaturated fatty acid (*n* = 10).
