# Supplementary figures and images for: Electroacupuncture at Acupoints Reverses Plasma Glutamate, Lipid, and LDL/VLDL in an Acute Migraine Rat Model: A 1H NMR-Based Metabolomic Study
Source: Evid Based Complement Alternat Med. 2014 Jan 28;2014:659268. doi: 10.1155/2014/659268 (PMC3921982; doi:10.1155/2014/659268)

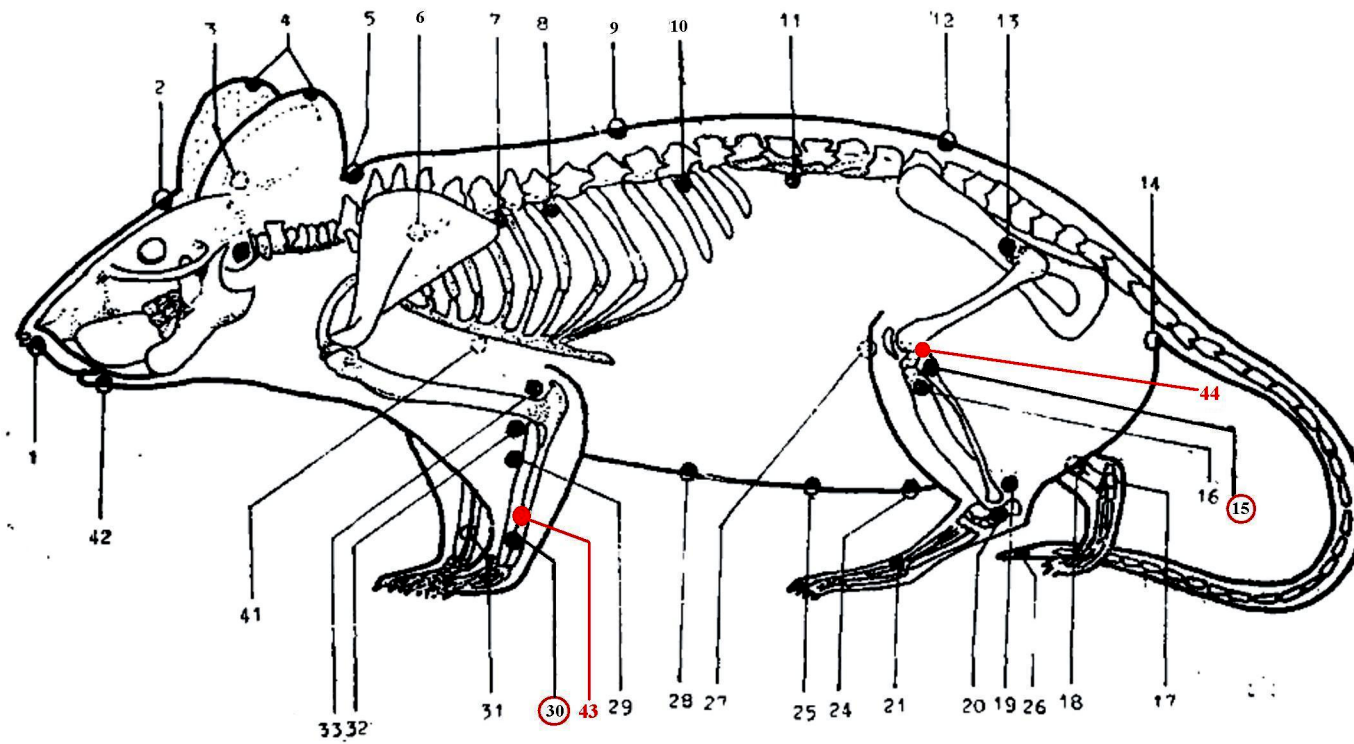

Supplement: Supplementary file 1 — Supplementary Figure 1: Anatomy of Rat Acupoints. Acupuncture treatment on NTG rats were conducted according to the picture of anatomy of rat acupoints. 15, Yanglingquan (GB34); 30, Waiguan (SJ5); 43-44, non-acupoints. Supplementary Figure 2: Imbalanced metabolism systems in NTG group. Imbalanced glycometabolism, lipid metabolism and amino acids metabolism were illustrated between NTG and control group. [file 659268.f1.pdf]

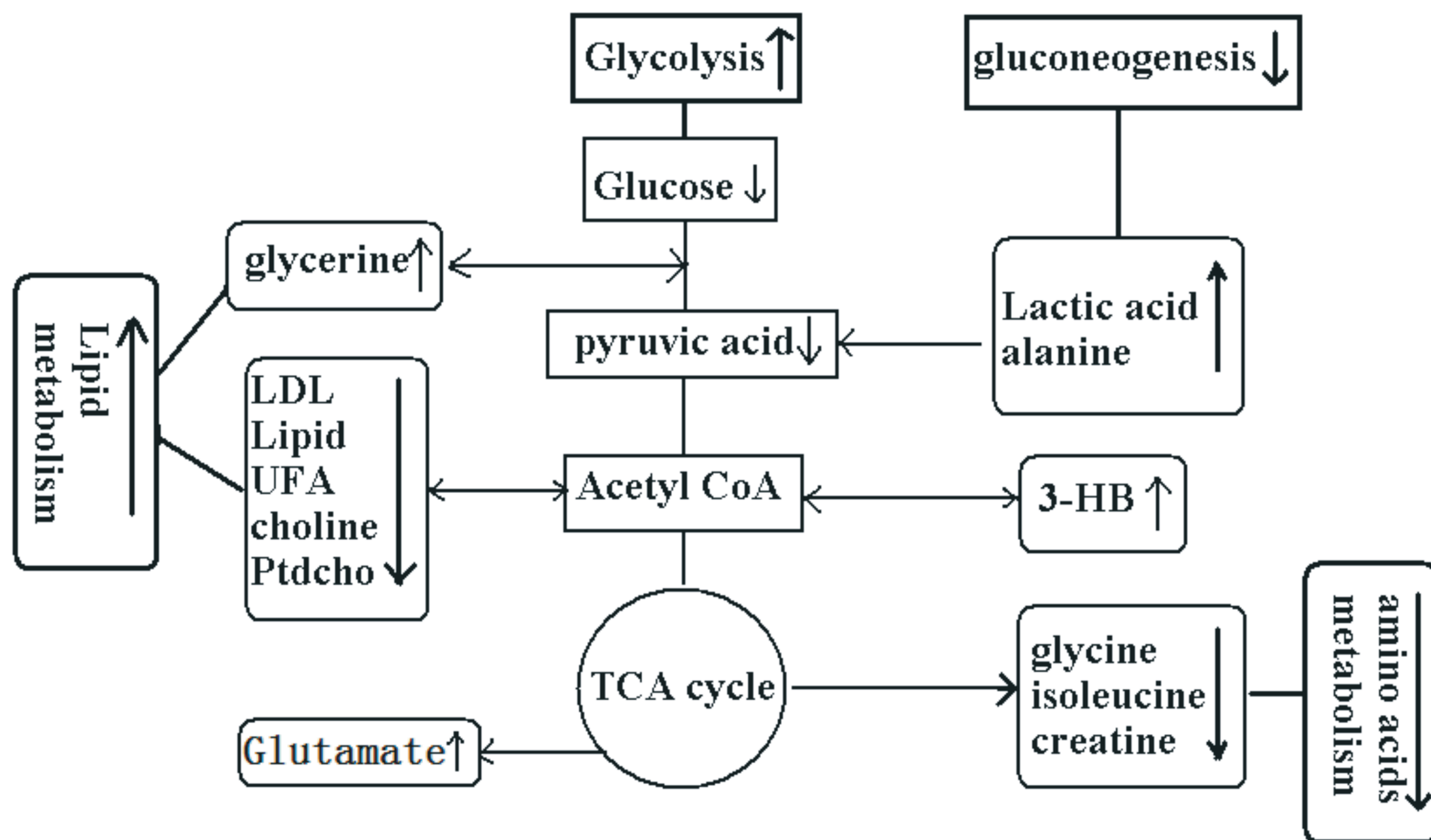

Supplement: Supplementary file 2 [file 659268.f2.pdf]
